# Involving People With Lived Experience in Electronic Health Record Database Studies Reflections and Learning From the CHOOSE Study

**DOI:** 10.1111/hex.70131

**Published:** 2024-12-18

**Authors:** Emma Cockcroft, Vidhi Bassi, Pearl L. H. Mok, Alex Adams, Anabel A. Claro, Alex M. Trafford, Matthew J. Carr, Darren M. Ashcroft, Emma Garavini, Rachel Temple, Roger T. Webb, Shruti Garg, Carolyn A. Chew‐Graham

**Affiliations:** ^1^ Department of Health and Community Sciences University of Exeter Exeter UK; ^2^ The McPin Foundation London UK; ^3^ Centre for Pharmacoepidemiology & Drug Safety, Division of Pharmacy & Optometry, Manchester Academic Health Sciences Centre The University of Manchester Manchester UK; ^4^ NIHR Greater Manchester Patient Safety Research Collaboration The University of Manchester Manchester UK; ^5^ Division of Psychology & Mental Health, Manchester Academic Health Sciences Centre University of Manchester Manchester UK; ^6^ Royal Manchester Children's Hospital Central Manchester University Hospitals NHS Foundation Manchester UK; ^7^ School of Medicine, Faculty of Medicine and Health Sciences Keele University Keele UK

**Keywords:** electronic health records, lived experience, mental health research, patient and public involvement and engagement (PPIE), young people, youth involvement

## Abstract

**Background:**

Patient and public involvement and engagement (PPIE) is integral to health research. Reporting of PPIE methods and impact is becoming increasingly common in health research. However, reporting on PPIE in studies using large, routinely collected electronic health record data sets is less common. Anecdotal evidence suggests that involvement in this research context is more challenging and offers fewer opportunities for meaningful influence on the research process.

**Objectives:**

This paper reports the involvement approach for a Clinical Practice Research Datalink (CPRD) study and critically reflects on the process and impact of involving young people, parents and carers in research using this UK primary care electronic health record data set.

**Methods:**

The CHOOSE study investigated mental health diagnoses of children and young people (1–24 years) during the COVID‐19 pandemic using the CPRD. The study was informed by a Lived Experience Advisory Panel (LEAP) which consisted of 13 members including 8 young people (13–25 years) with lived experience of mental health difficulties and 5 parents/carers, with involvement activities facilitated by project partners, mental health research charity, The McPin^R^ Foundation. We reflect on this process in this manuscript.

**Results:**

Key benefits of involving people with lived experience in this research included making sense of and contextualising findings and ensuring that they were focused on making a difference to young people's lives. Challenges included the fixed nature of the CPRD data, which did not capture all the information people with lived experience perceived to be important. Researchers expressed limited time for PPIE activities although that was compensated by McPin colleagues who organised and facilitated online meetings, and supported the young people, parents and carers during and between meetings.

**Conclusions:**

This paper describes an approach to patient and public involvement in an electronic health record database study. Working collaboratively with young people, carers and other stakeholders requires sufficient time and adequate resources. We also highlight the importance of appropriate training and support and being transparent about the limitations of PPIE involvement.

**Patient or Public Contribution:**

Three members of the CHOOSE LEAP have been involved in conceptualising and writing this paper.

## 1 Introduction

Patient and public involvement and engagement (PPIE) in designing health care services and research is essential [1, 2, 3, 4]. It entails an active partnership between members of the public and researchers. This means that members of the public with relevant lived experience of the research topic work alongside the research team; they are actively involved in contributing to the research process as advisors and possibly co‐researchers [5]. PPIE ensures that research is more relevant, acceptable, and appropriate for those whom it intends to affect. Furthermore, the active involvement of patients and members of the public in setting research agendas ensures that research is aligned with the priorities and needs of those affected. This co‐production approach increases the relevance and impact of the research, minimising the risk of pursuing redundant topics. This alignment between research objectives and stakeholder priorities can significantly reduce avoidable waste in the production and reporting of research evidence [6].

A number of models and frameworks have been developed for supporting PPIE approaches in diverse activities, such as research agenda setting [7], clinical trials [8], drug development [9], as well as the production and reporting of research [10]. In addition, guidance is available from organisations such as the National Institute for Health and Care Research (NIHR) [11] in the United Kingdom and the Patient‐Centred Outcomes Research Institute (PCORI) [12] in the United States. These guidelines primarily concern one‐directional involvement strategies where researchers involve patients and public contributors. Adapting the general guidance to specific research situations can be challenging [13, 14, 15].

In studies where people with lived experience had no clear role or felt only sporadically involved [15] their participation may be interpreted as tokenism [16], and can be experienced as ‘virtue signalling’ [17]. It has been suggested that weak empirical evidence about the impact of PPIE in research leads to a lack of consensus about what constitutes effective involvement processes [18].

A review identified 65 frameworks for PPIE in research [19]; one factor highlighted as important was encouraging the involvement in *all* research phases, starting from the project formation phase. Furthermore, creating a culture of continuous involvement and developing relationships and trust were emphasised together with building understanding about the local context and offering opportunities for training and capacity building. Challenges that were identified include the use of different terminology, lack of knowledge of specific research issues, and health‐related challenges in life that set boundaries for lay participants’ research involvement [19].

Although PPIE is now a common feature in research across the UK with examples of PPIE in reviews [20] and in doctoral research projects (e.g., PhD dissertations), ^21^its implementation in studies that rely on quantitative research methods, particularly those using routinely collected large‐scale data, remains less frequently reported and under‐explored [21, 22, 23, 24]. Previous work has focused on reporting an analysis of PPI statements in methodology sections of published papers, one‐off involvement activity or statistical analysis. As researchers experienced in facilitating patient and public involvement in health research, we have anecdotal evidence of different researcher perspectives of the scope of involvement in this type of research. This indicates that involvement is more difficult with less scope for influencing the conducted investigation. It is therefore important to provide a reflective account of how to involve patients in studies that are conducted using large routinely collected electronic health record data sets and the potential for influencing the research and the challenges that thereby arise.

## 2 Aim

To critically reflect on the processes, challenges and opportunities in involving young people, parents and carers in a study conducted using an electronic health record data set.

## 3 Involvement Context

The CHOOSE study (CHildren and yOung peOple pSychiatric diagnoses before and during the COVID‐19 pandEmic) aimed to investigate mental health diagnoses and self‐harm episodes among children and young people in UK primary care records before and during the COVID‐19 pandemic). The study used data from the Clinical Practice Research Datalink (CPRD), a database of anonymised electronic health records sourced from over 2000 general practices covering around 25% of the UK population. We sought to provide recommendations for young people, parents and for health and social care, educational and VCSE (Voluntary Community Social Enterprise) services about how to support the mental health of children and young people following the pandemic. The study investigated how many children and young people (aged 1–24 years) were diagnosed with a mental health problem or have had self‐harm recorded by GPs since the start of the pandemic in March 2020 and how these numbers compared to those observed before the pandemic. Statistical models were used to predict incidence rates for the medical health conditions of interest to enable comparison between the expected rates based on previous trends and the observed incidence to give an indication of the changes since the onset of the pandemic.

The study was shaped by the involvement of two groups:

- Lived experience advisory panel (LEAP): 13 members including 8 young people (13–25 years) with lived experience of mental health issues and 5 parents/carers.
- Stakeholder advisory group: 15 members including teachers, healthcare professionals, third sector practitioners with an interest in young people's mental health.

Although we worked closely with both of these groups, the focus of this paper is to describe and reflect on the involvement of the LEAP.

## 4 Involvement Methods

Involvement of the LEAP was co‐ordinated by the McPin Foundation^R^ in collaboration with other members of the research team, including those with expertise in facilitating involvement and engagement activities. McPin^R^ is a mental health research charity focused on transforming mental health research through the involvement of expertise of people with lived experience.

### 4.1 Recruitment

Recruitment of LEAP members was through several approaches including social media, advertisements in McPin^R^ Young People's Network and websites, and through contacting existing networks and organisations known and with established relationships to McPin^R^. Recruitment material gave details of the project, what being involved would require, support that would be provided and details of reimbursement. The recruitment approach was planned considering who needed reaching and how best to do this, with a focus on reaching parents and carers. Although formal informed consent was not required for this PPIE activity, LEAP members were given information about what would be involved and their ability to leave the LEAP at any point without consequence. Each LEAP member had to complete an emergency contact form before beginning their involvement and parental consent was required for those under 18. Each member was provided with the McPin^R^ advisory group information sheet to provide full details about their role, requirements, support and payment.

### 4.2 Training and Support

Members of McPin^R^ met with all LEAP members individually to discuss involvement and any individual accessibility requirements or support needs before the start of the project, such as mental health and neurodiversity support needs, and provision of IT equipment to ensure that members were not digitally excluded. Additional support was given before and after meetings, along with regular email contact and updates throughout the project. Members of the McPin^R^ team were available for support via email or phone to young people and carers when needed. Through working with the LEAP and understanding their needs, appropriate training was identified, and included.

### 4.3 Reimbursement

Panel members were paid £50 for participation in a 2‐h meeting. For ad hoc and written input in between meetings or involvement in additional dissemination activities panel members were paid £25 per hour. Payment was co‐ordinated via invoice to McPin^R^. Payment via vouchers was also provided as an option for panel members if they preferred. Support was available for completing forms, with reminders sent if members had not returned invoices.

### 4.4 Involvement Format

#### 4.4.1 Group Meetings

Involvement of the LEAP was through online meetings, email, and input through live documents and Google forms and polls for voting exercises. The details below reflect the involvement during the project's main phases, with ongoing work in developing dissemination materials and writing research papers.

In total six online meetings were held during the study (see Table 1). These were spread throughout the project's 19‐month duration with each meeting having a clear specific focus. Although areas of input were planned beforehand there was flexibility to address emerging issues. The dates and meeting purpose are shown in Table 1. Meeting length was between 1 and 2 h depending on the agenda. Shorter 1‐h meetings enabled us to maintain engagement with the group during periods when researchers were analysing data. All meetings were attended by members of the research team to help ensure integration of discussions into the data analysis and interpretation of findings. Meetings were facilitated by members of McPin^R^. Every meeting began with the PPIE facilitator going through the agenda and the ground rules for the meeting. This was then followed by an icebreaker activity that was unrelated to the research topic as a means of making everyone in the meeting feel comfortable, building rapport and for the group to get to know one another outside of their named roles, including the academic members of the team. Where appropriate virtual breakout rooms were used to allow for smaller group discussions. Notes were made and distributed after meetings, including a ‘you said, we did’ table. This approach helped to ensure integration of LEAP perspectives, as well as providing transparency and feedback to the groups as to how researchers responded to discussions. Input was also received outside of formal meetings. This included commenting on an initial project protocol, any post‐meeting reflections, feedback for future meetings, comments on documents and input via polls for deciding on the acronym for the study name, and in what format and how often group members wanted to be contacted.

**Table 1:** LEAP project meeting dates and purpose.

Meeting # and date	Purpose
#1: Jan 2022—2 h meeting	Introduction to project, initial questions and next steps
#2: May 2022—1 h meeting	Introduction to CPRD data and data analysis^a^ Using records of fictitious patients to illustrate the CPRD data.
#3: July 2022—1 h meeting	Discussion of preliminary findings
#4: Oct 2022——2 h meeting	Update and further findings, key questions and discussion of implications
#5: Feb 2023 – 2 h meeting	Dissemination plan discussion
#6: March 2023—2 h meeting	Consensus group meeting including members of the stakeholder advisory group to discuss: What are the key messages? Who should the messages be aimed at? Implication of findings What should the output be.

a This was an extra meeting stemmed from discussions during the first meeting when it became apparent that, for members of the LEAP to truly contribute to the study, a meeting solely to describe the database was needed.

#### 4.4.2 Dissemination and Additional Activity

Members of the advisory group were involved in several of the dissemination outputs, with their contributions ensuring that the outputs were shaped for and reached the appropriate audiences. Members were able to indicate the dissemination activities that they wished to be involved in.

A video titled ‘what to do if you think you have a mental health problem’, shaped by two members of the youth advisory group was a dissemination output targeted at young people. The two advisory group members wrote and/or gave input on the script and animation and approved final edits (https://youtu.be/2sB5zu5fQ7Q). This was done via Google Forms and email feedback. Four infographics summarising the key findings and messages targeting different audiences, young people, parents/cares, clinicians and teachers, were produced with involvement from four members of the advisory group. The members shaped and edited the text and input into the design, format and sharing plans for each of the four infographics. This was done via Google Forms and email feedback. Three members of the advisory group have co‐authored this paper. Their input shaped the content and focus of the paper as well as providing reflections on their experience of being an advisory group member and co‐author. Their involvement in this paper has been via several meetings with some of the authors of this paper, written work and input in between meetings via Google Docs and Word Documents with tracked changes and email input.

**Figure 1:**
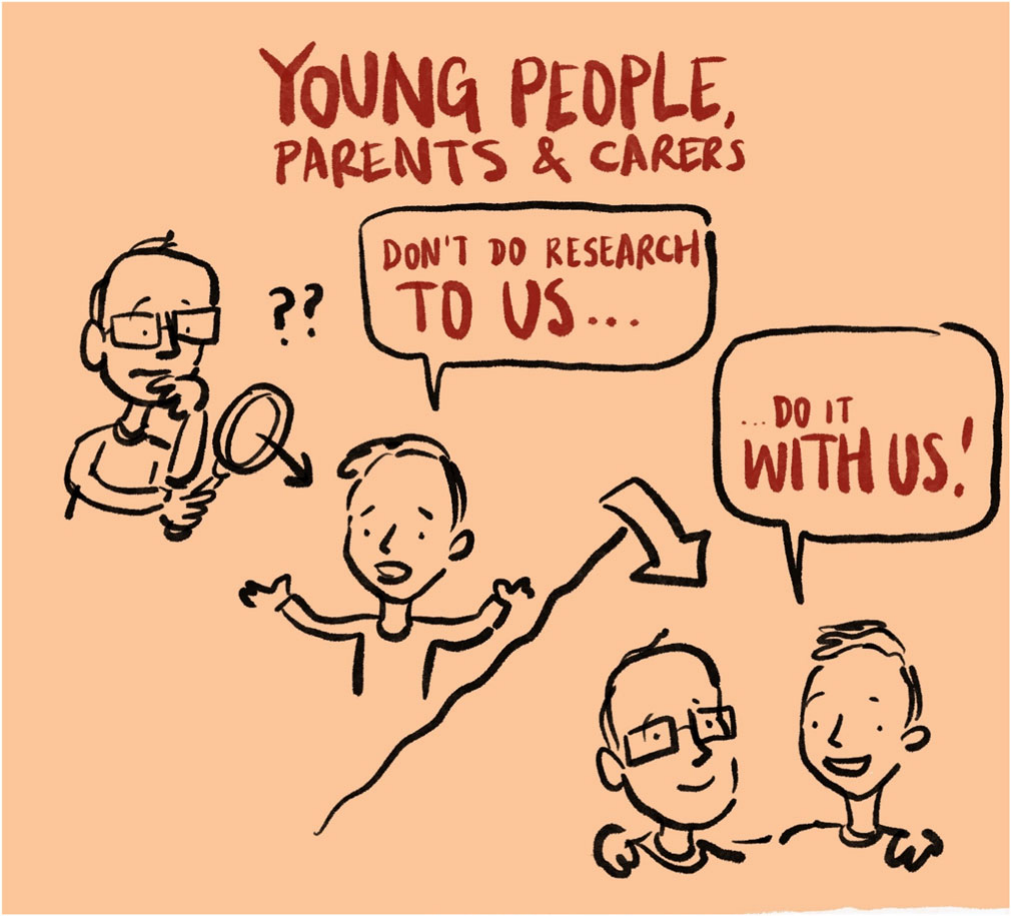
Example illustration created during live webinar.

An end of project webinar, co‐chaired by a young person advisory group member, with reflections from young people and parents, was a key dissemination activity. All advisory group members involved received support for the webinar with script writing and practise sessions with McPin^R^ staff member (E.G.) via 1‐1 meetings, email support, as well as a final practise session with all project team members involved in the webinar. The webinar was illustrated live and illustrations used for other dissemination activities (see Figure 1).

## 5 Patient and Public Involvement

Alongside the involvement of LEAP members directly to the CHOOSE study, we have worked closely with three members of the CHOOSE LEAP and McPin^R^ staff in conceptualising and writing this paper. We held four online meetings where early ideas were shared and discussed, and the process of involvement in the CHOOSE study was reflected upon. Drafts of the paper were circulated and edited for editing and feedback with the LEAP members throughout the writing process.

## 6 Reflections and Learning

### 6.1 Analytical Approach

In alignment with previous PPIE research, we incorporated a reflexive approach where members of the research team, LEAP, and McPin^R^ engaged in structured reflective discussions. These discussions were centred on questions such as: ‘What impact did LEAP input have on decision‐making?’, ‘What barriers were encountered?’, and ‘What strategies could enhance PPIE in similar future studies?’ These reflections were used to develop a deeper understanding of the nuances of conducting PPIE in routine database studies.

By employing this analytical and reflective framework, this study provides a nuanced account of PPIE in large‐scale database research, addressing both methodological challenges and practical considerations for future projects.

### 6.2 Key Reflections and Learning

Several key reflections were made relating to the benefits and challenges of involving people with lived experience in studies conducted using large routinely collected electronic health record data sets, as well as reflections on how the involvement process could be improved.

### 6.3 Training and Support

After discussions during the first online meeting it was clear that training around CPRD would be helpful and, indeed, necessary to maximise potential of young people's involvement. A reflection from one of the LEAP members on this can be found in Box 1. The fixed nature of CPRD data, which are collected routinely for administrative rather than research purposes, and the stringent epidemiological study design and methodological parameters meant that there was little scope for the research team to incorporate suggestions from the LEAP members about the study design and areas of investigation. As a result of early discussions we incorporated training on the data set (where the data came from, how they were collected, what they covered and their limitations) and methods of analysis into a subsequent meeting (meeting 2, Table 1), which LEAP members found helpful.

Box 1: **Reflection of Leap Member on Training and Support**

‘Throughout my involvement in the project I felt prepared, engaged with the rest of the team, and able to contribute across a variety of tasks. The support on offer was thoroughly thought out and heavily emphasised which was a nice touch. It has been a fantastic insight into how research can be done’. LEAP member and paper co‐author

LEAP members reported that they felt supported throughout the project, with briefings before and after meetings and email updates and check‐ins throughout enabled them to feel part of the process. It was felt that staff were available and if in doubt they could be emailed or phoned. This support also related to reimbursement and payment for involvement. There was help available for submission of invoices and administrative help with forms making it easy to make claim for payment. There was an adaptable system in place to meet individual needs. There was a dedicated member of staff on the project who co‐ordinated all LEAP activities. This meant that LEAP members had a ‘go to’ contact, with the member of staff specifically responsible for ensuring good practice for meaningful involvement. Involvement in dissemination activities also required extra training and advice, in particular supporting LEAP members involved in the project webinar (and co‐chairing this), infographics and the video.

### 6.4 Recruitment to LEAP

The process of finding a wide range of people with relevant lived experience in involvement in research is not often reported. In this project it was noted how difficult it can be. McPin^R^ used a thought through recruitment strategy, tailoring recruitment to different audiences and with proactive consideration as to where we would find people and how. Recruitment material was carefully designed to catch the eye and encourage people to be involved, with the details of the role and what to expect fully outlined. Notably, people who identify as young males, from low‐income backgrounds, LGBTQ+ and from ethnic minorities are often underrepresented in involvement [25]. Text was included that explicitly encouraged such groups to apply, and places on the group were actively reserved for individuals with this expertise. McPinR invested significant resources in planning and facilitating appropriate recruitment to the group, with recruitment open for a 1 month period, with regular social medial posts and emails to other organisations as well as internal and external networks. A reflection from one of the LEAP members on this can be found in Box 2.

Box 2: **Reflection of Leap Member on Recruitment**

‘I came across the opportunity via the McPin's Newsletter and found that the advertisement outlined the inclusion criteria clearly, which helped me reflect on whether I was a suitable candidate. There was enough time given to submit the application, and I appreciated the opportunity to talk to McPin staff directly about my doubts before applying. It made me felt confident that this was the right opportunity for me’. LEAP member and paper co‐author

Recruitment of parents and carers to the advisory group presented challenges due to not many parents/carers applying to join the group initially. McPin identified this by regularly checking the applications from people during the 1‐month period that recruitment was open. Upon identifying a small number of applications from parent/carers, McPin staff tailored recruitment efforts to reach these groups by sending out the advert opportunity to their wider networks, existing groups of adult advisory groups attached to McPin and contacting partner organisations with parent/carers as their key audiences.

LEAP members felt encouraged by the clear messages and knew who they were looking for and what would be involved. The early conversations with the McPin team made it clear to LEAP members that support would be in place in relation to support with mental health, accessibility, neurodiversity etc., which was reassuring.

### 6.5 Impact and Benefits

Several benefits of being involved were discussed. These included personal benefits and enhancement of the research itself. From the perspective of young people and carers involved in the project, there was a sense of being able to relate to others’ experience and work as a team, and feeling positive about being able to contribute to something meaningful Bringing people together from different backgrounds, but with the same passion to collaborate on a shared goal, was also reportedly important. Those involved also welcomed the opportunity to be part of the consensus group exercise (meeting #6: March 2023), which brought together the LEAP and the stakeholder group. Members also valued being able to inform future recommendations for youth mental health, which gave a sense of achievement in completing the project. LEAP members also had the opportunity to develop other useful skills. For example, the LEAP member who co‐led the end of project webinar reported gaining confidence from this opportunity. Career development opportunities such as co‐authorship of research papers was also an important aspect of the work. A reflection from one of the LEAP members on this can be found in Box 3.

Box 3: **Reflection of Leap Member on Impact and Benefit**

‘It is great to see the outcomes of the teams work and wonderful particularly to have contributed to this as, of course, it is something that is very important to me, so working in this area has been incredibly rewarding’. LEAP member and paper co‐author ‘I had an amazing opportunity to collaborate with others who are passionate about the same topics as me and have similar experiences to mine. Through this experience, I gained a greater insight into the research process and honed my research skills. I believe it was incredibly beneficial that the research team involved us in the dissemination efforts. As young people, we brought a unique perspective that helped ensure the content was not only highly relevant but also engaging to our demographic’. LEAP member and paper co‐author

In terms of impact on the research, meaningful and integrated involvement helped to make sense of and contextualise the findings from the project. It steered the focus towards making a difference to young people. For instance, involvement helped to define the study questions, interpret results, identify key messages and formulate disseminate plans in what is often deemed a fairly fixed research process. For example, members gave insights into the possible reasons for the sex‐differences observed in eating disorders and in self‐harm incidence and how the use of social media could have influenced the mental health of males and females differently. They provided possible explanations as to why the mental health of adolescent girls was particularly adversely affected during the pandemic and the socio‐economic differences observed. By involving the LEAP members in the dissemination planning and activities, we were able to ensure that the key messages and their targeted audience were relevant, and the dissemination channels were optimal. One researcher initially expressed doubt as to the value of PPIE in a CPRD study given that the data were collected routinely and prospectively, so we could not influence what data to collect, but reflected on how useful and valuable the LEAPs input has been.

### 6.6 Challenges and Recommendations

Through reflecting on the process, we have identified several challenges to meaningful involvement in this type of research. These identified challenges, along with recommendations for future projects, are outlined in Table 2.

**Table 2:** challenges and recommendations for the involvement of people with lived experience in large database research.

Challenges	Recommendations
Fixed nature of database studies	Training early in involvement process to ensure all are aware of the limitations and restrictions of the data and hence their influence on the study design and scope. This helps to manage expectations of those involved in terms of what is and isn't possible to change.
Training needs	Training and support needs can vary depending on group members and specifics of the project. Having the ability to respond to the wants and needs of the group can help to build meaningful input.
Being reactive to dissemination suggestions	Build flexibility into grant applications. Ensure dissemination and impact activities are specifically considered when budgeting for projects including staff time to facilitate and support.
Time and resource requirement	Ensure appropriate costings in funding applications to allow for flexibility within the involvement process as well as adequate support costs.
	Consider working with charities and external organisations to help support and facilitate involvement

One key challenge was that the fixed nature of routinely collected electronic health record data sets. This was in terms of limitations in what data are collected and what questions can be answered, meaning that much of the first meeting felt like we could not take on board important suggestions and LEAP members feeling unheard. For example, we were not able to include ethnicity in our analyses as suggested by the members because of the amount of missing data in the CPRD primary care records. It was felt that if training was provided at an earlier stage of the process, then this could help to manage expectations of what is and isn't possible. The need for provision of sufficient training has previously been reported and suggested that it may also help to foster positive working relationships [26]. Training should be context specific, in the case of CPRD projects, this would include details about the composition of the data set and its strengths and limitations. We were able to respond to LEAP members uncertainties and provide specific training to address their concerns and needs. Additionally, it is worth noting that the fixed nature of this type of data may be one of the reasons for the apparent lack of PPIE in this type of research. Alongside provision of training for patients involved in CPRD projects it is also worth noting the importance of clear suggestions to research as to how patients can still have impact and be meaningfully involved.

An additional challenge that we encountered was the ability to be reactive to suggestions, particularly around dissemination activities. The need to be preemptive in funding applications in terms of appropriate costings and support can limit flexibility within involvement activities. During discussions about who to disseminate to and how, it was agreed that several approaches were needed, which had not been accounted for. For these aspects to be co‐produced, we needed to find more funding to enable payment of young people and carers involved and to provide support for these activities from McPin^R.^—support for individuals involved is also invaluable for meaningful involvement (which has additional cost implications). In this project we worked closely with McPin^R^ who added invaluable expertise in working with young people and carers with experience of mental health issues. The need for appropriate costing for this support has been previously emphasised [27, 28] and our reflections reiterate the importance of working closely with organisation to help facilitate involvement. For the CHOOSE project PPIE funding accounted for ~20% of the total spend. This was originally 15% of the budget, with additional funding being sought to allow for additional input from LEAP members. When budgeting for PPIE activities we suggest the importance of specifically considering dissemination, impact and influence and building flexibility into project plans.

## 7 Conclusion

We describe the process of and reflections on the involvement of young people and carers with relevant lived experience in a database study investigating mental health diagnoses and self‐harm episodes among children and young people in UK primary electronic health records before and during the COVID‐19 pandemic.

This work contributes to the evidence base by providing a practical example of enabling meaningful involvement in studies that use routinely collected electronic health records. We recommend that researchers using routine data sets consider early engagement, transparent communication about data limitations, and the inclusion of PPIE‐specific training that addresses the unique constraints of routine data analyses. Addressing these considerations can help maximise the value of PPIE in this context and ensure that contributions are meaningfully integrated

We recommend partnering with charities or organisations with experience of working with specific population groups to help integrate and facilitate involvement throughout the process, with dedicated staff to facilitate involvement activities and support those involved. Additionally including training early on in the process that is context‐specific on research methods and scope/limitations of a study helps to avoid the potential of those involved feeling like they are not being heard and to build trust. From our perspective the involvement of people with lived experience in projects conducted using routinely collected data sets is essential and of benefit to the research, the researchers and those involved.

## Author Contributions

**Emma Cockcroft:** conceptualisation, investigation, funding acquisition, writing–original draft, methodology, writing–review and editing. **Vidhi Bassi:** investigation, writing–original draft, methodology, writing–review and editing. **Pearl L. H. Mok:** conceptualisation, investigation, funding acquisition, writing–original draft, methodology, writing–review and editing. **Alex Adams:** investigation, writing–original draft, methodology, writing–review and editing. **Anabel A. Claro:** investigation. writing–original draft. methodology. writing–review and editing. **Alex M Trafford:** writing–original draft, methodology, writing–review and editing, conceptualisation. **Matthew J. Carr:** conceptualisation, investigation, funding acquisition, writing–original draft, writing–review and editing; methodology. **Darren M Ashcroft:** conceptualisation, investigation, funding acquisition, methodology, writing–review and editing. **Emma Garavini:** investigation, writing–original draft, methodology, writing–review and editing. **Rachel Temple:** investigation, writing–original draft, methodology, writing–review and editing. **Roger T. Webb:** conceptualisation, investigation, funding acquisition, writing–review and editing, methodology. **Shruti Garg:** investigation, funding acquisition, writing–review and editing.

## Conflicts of Interest

C.C.G. is editor‐in‐chief of Health Expectations. The other authors declare no conflicts of interest.

## Data Availability

Data sharing is not applicable to this article as no data sets were generated or analysed as part of this report.
